# Correction to “ELF3‐AS1 contributes to gastric cancer progression by binding to hnRNPK and induces thrombocytosis in peripheral blood”

**DOI:** 10.1111/cas.16318

**Published:** 2024-08-19

Song S, He X, Wang J, et al. ELF3‐AS1 contributes to gastric cancer progression by binding to hnRNPK and induces thrombocytosis in peripheral blood. Cancer Sci. 2021; 112: 4553–4569. 10.1111/cas.15104

There was an error in Figure 4(E) BGC 823 group. Here is the corrected Figure 4(E) BGC 823 group.

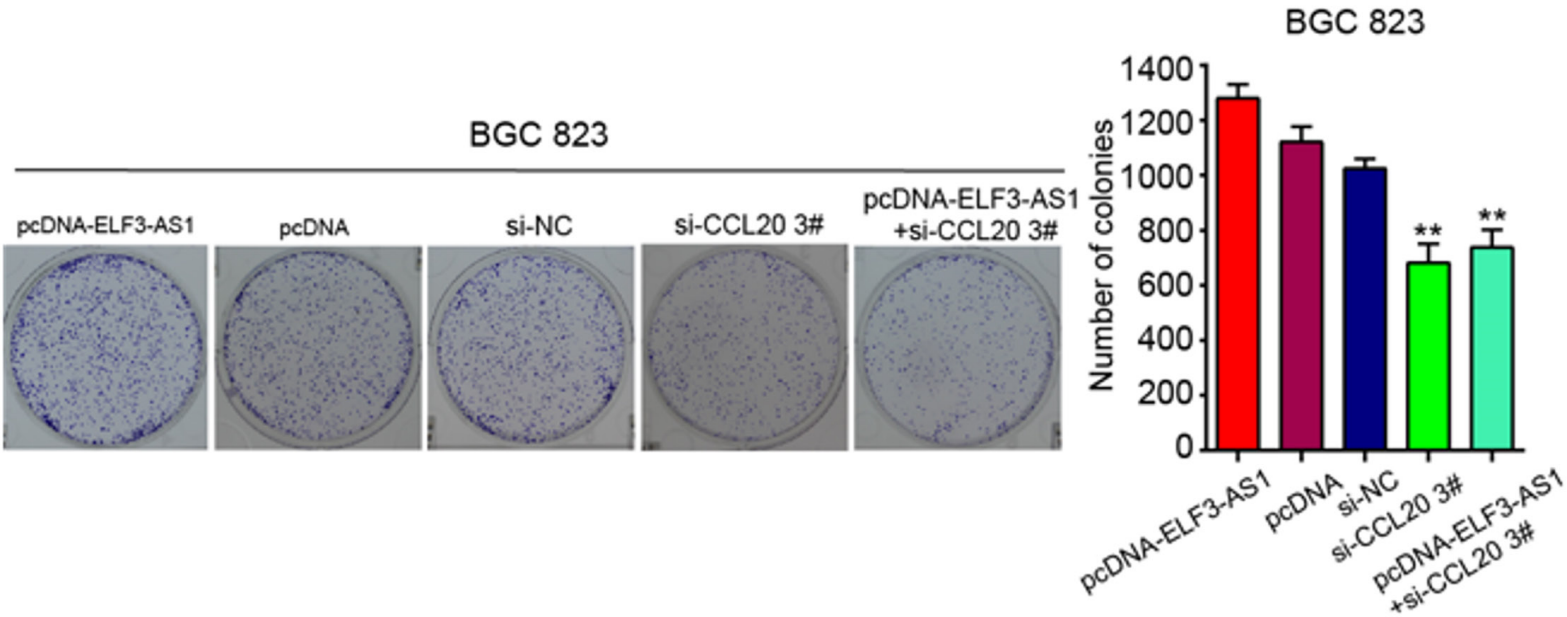

We apologize for this error.

